# Metastasis of Melanoma to the Adrenal Glands: A Case Report and Literature Review

**DOI:** 10.7759/cureus.26749

**Published:** 2022-07-11

**Authors:** Asad A Haider, Ariel Ruiz de Villa, Leora Frimer, Yvette Bazikian

**Affiliations:** 1 Internal Medicine, University of Central Florida College of Medicine & Hospital Corporation of America (HCA) North Florida Regional Medical Center, Gainesville, USA

**Keywords:** inferior vena cava tumor thrombus, right atrial cardiac mass, braf mutation, cancer immunotherapy, metatatic skin cancer, ­skin cancer, adrenal disorders, bilateral adrenal masses, malignant melanoma metastasis

## Abstract

Immune checkpoint inhibitors have significantly improved the prognosis of metastatic melanoma, but metastases to the adrenal glands remain highly resistant to these new treatments. Adrenal gland metastases from melanoma can present in an unusual manner, such as in this report, making it diagnostically and therapeutically challenging. In this case report, we present a patient with histologically confirmed metastatic melanoma to the adrenal glands, a large intracardiac mass suspicious for metastatic disease, and an inferior vena cava thrombus. We review the existing literature to explain the unique characteristics, clinical relevance, pathogenesis, diagnosis, and treatment of adrenal gland metastases from melanoma.

## Introduction

Malignant melanoma is an aggressive proliferation of melanocytes arising from the skin, most commonly metastasizing to the liver, bone, and brain. The prognosis of metastatic melanoma has improved considerably in recent years with the use of targeted therapeutic agents, particularly immune checkpoint inhibitors (ICIs) [1]. Despite the new advances in treatment, adrenal metastases from melanoma are unique in that they remain highly resistant to ICIs [2,3]. The true incidence of adrenal involvement from primary cutaneous melanoma is unknown, and little data exists regarding the natural history of patients with this condition [4]. We describe a 70-year-old female presenting with dyspnea, who was found to have histologically confirmed bilateral adrenal gland metastases from malignant melanoma, as well as a large intracardiac mass clinically suspicious for cardiac metastasis and a thrombus in the inferior vena cava (IVC) clinically suspicious for tumor thrombus. Her case highlights the unique characteristics and clinical relevance of adrenal metastases from melanoma, as well as the need for more medical education about its pathogenesis, diagnosis, and treatment.

## Case presentation

The patient reported is a 70-year-old female with a past medical history of hypertension, type 2 diabetes mellitus, hyperlipidemia, and depression, who presented to the emergency department with a two-to-three-week history of dyspnea and generalized weakness. She endorsed exertional dyspnea with most activities of daily living, including walking, which was only alleviated by rest. Additionally, the patient reported chronic fatigue lasting months, as well as an unintentional weight loss of thirty-five pounds. She initially attributed her symptoms to depression, but ultimately believed this was unlikely as her chronic depression was controlled. The patient denied syncope, chest pain, abdominal pain, fevers, nausea, vomiting, night sweats, or any recent illness or trauma. 

The surgical history included a total hysterectomy for the treatment of uterine fibroids and the removal of lipoma from the abdomen. She denied any alcohol, tobacco, or recreational drug use. Family history was pertinent for relatives having hypertension, coronary artery disease, and leukemia, but there was no family history of any other malignancies. The patient was in the process of retiring and did volunteer work for a nonprofit organization. The patient took the following home medications once a day: metformin 500 mg, bupropion 150 mg, sertraline 100 mg, atorvastatin 20 mg, and a combination of benazepril and hydrochlorothiazide 10/25 mg. Vital signs in the emergency department were stable. A comprehensive physical examination was normal. In the emergency room, the patient underwent testing for a comprehensive metabolic panel (CMP), complete blood count (CBC), coagulation panel, troponin level, and N-terminal-prohormone brain natriuretic peptide (NT-proBNP). Abnormalities seen on initial lab studies were an aspartate aminotransferase (AST) value of 67 units/L (normal range 15-37 units/L), an alkaline phosphatase level of 280 units/L (normal range 38-126 units/L), and a hemoglobin level of 10.3 g/dL (normal range 11.2-15.7 g/dL). All lab abnormalities were chronic and were at approximately the patient’s baseline levels. All other lab values were within the normal range.

**Figure 1:**
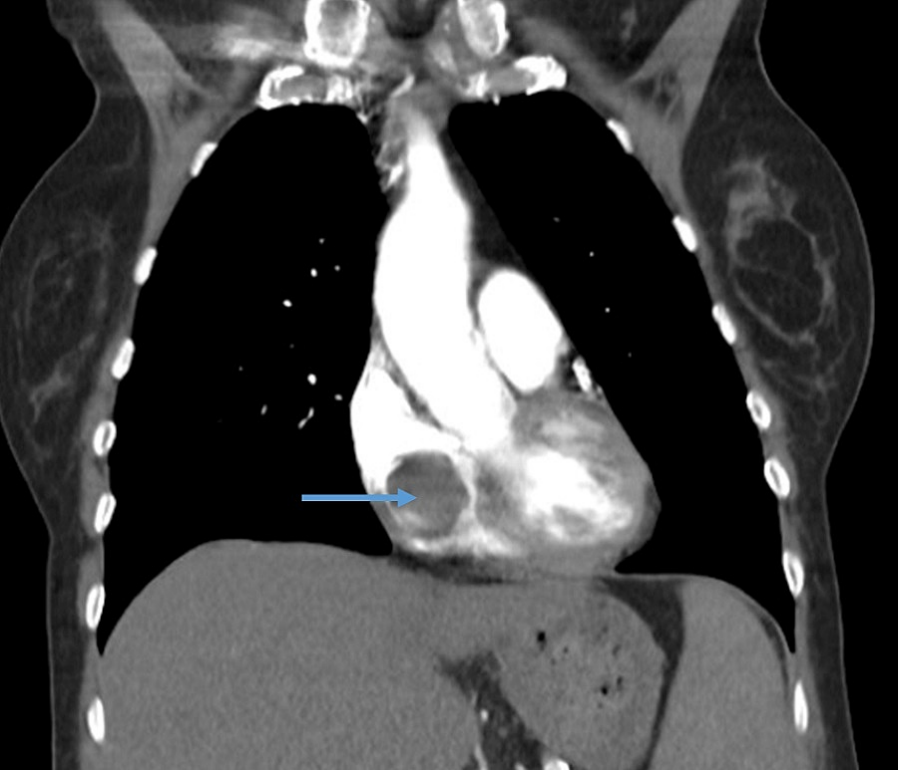
Representative CTA image of the chest showing a heterogeneous mass in the right atrium of the heart. Blue arrow indicates the location of the mass. CTA: computed tomography angiography.

**Figure 2:**
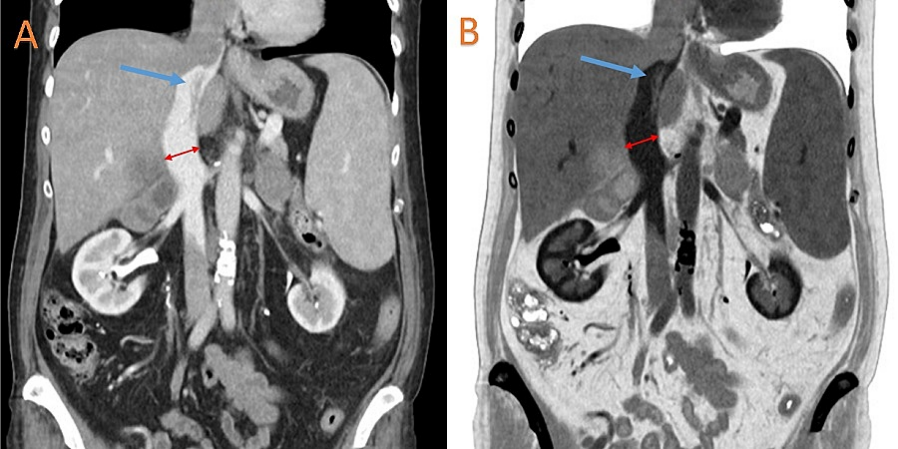
Representative original image (A) and inverted image (B) of the CT of the abdomen showing a thrombus extending from the IVC to the hepatic veins. Blue arrows indicate the location of the thrombus in the IVC. Red arrows indicate vessel expansion of the IVC due to the thrombus, suggesting a tumor thrombus rather than a bland thrombus. CT: computed tomography. IVC: inferior vena cava.

**Figure 3:**
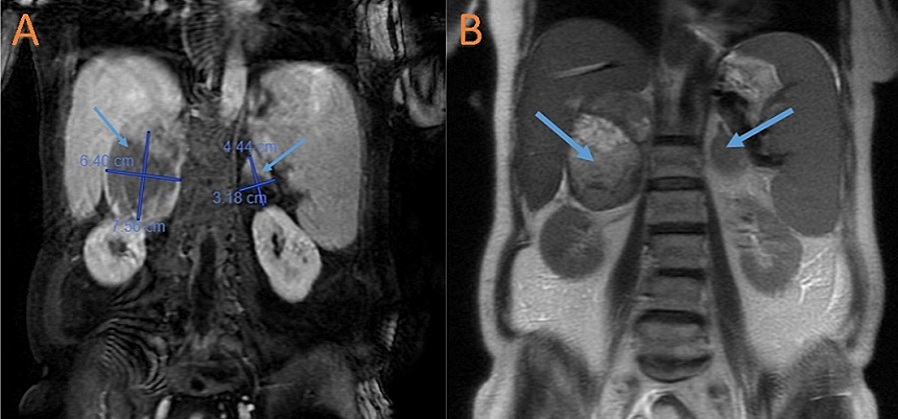
Representative original image (A) and inverted image (B) of the MRI of the abdomen showing bilateral, heterogeneous adrenal gland masses. Blue arrows indicate the location of the adrenal gland masses, with accompanying measurements of the masses in (A). MRI: magnetic resonance imaging.

A computed tomography angiography (CTA) scan of the chest revealed a 4.5 cm heterogeneous mass within the right atrium of the heart (Figure 1). A computed tomography (CT) scan of the abdomen revealed a large thrombus extending from the hepatic veins to the IVC (Figure 2). Vessel expansion of the IVC due to the thrombus suggested tumor thrombus rather than bland thrombus. A magnetic resonance imaging (MRI) scan of the abdomen revealed large bilateral heterogeneous adrenal masses, the right-sided mass measuring 7.56 × 6.40 cm and the left-sided mass measuring 4.44 × 3.18 cm, with no lymphadenopathy (Figure 3).

The patient was immediately started on a continuous infusion of intravenous heparin and admitted to the hospital. Specialists from cardiology, interventional radiology, and hematology-oncology were consulted to participate in the assessment and treatment of this patient alongside the internal medicine service. As the patient was hemodynamically stable, no procedural intervention was recommended with regard to the large clot burden in the IVC. A brewing malignancy involving multiple systems was suspected. 

On further history gathering, the patient denied any history of hormone replacement therapy, blood clots, bleeding disorders, or malignancy. She reported normal mammograms, colonoscopies, and cervical cancer screenings.

**Figure 4:**
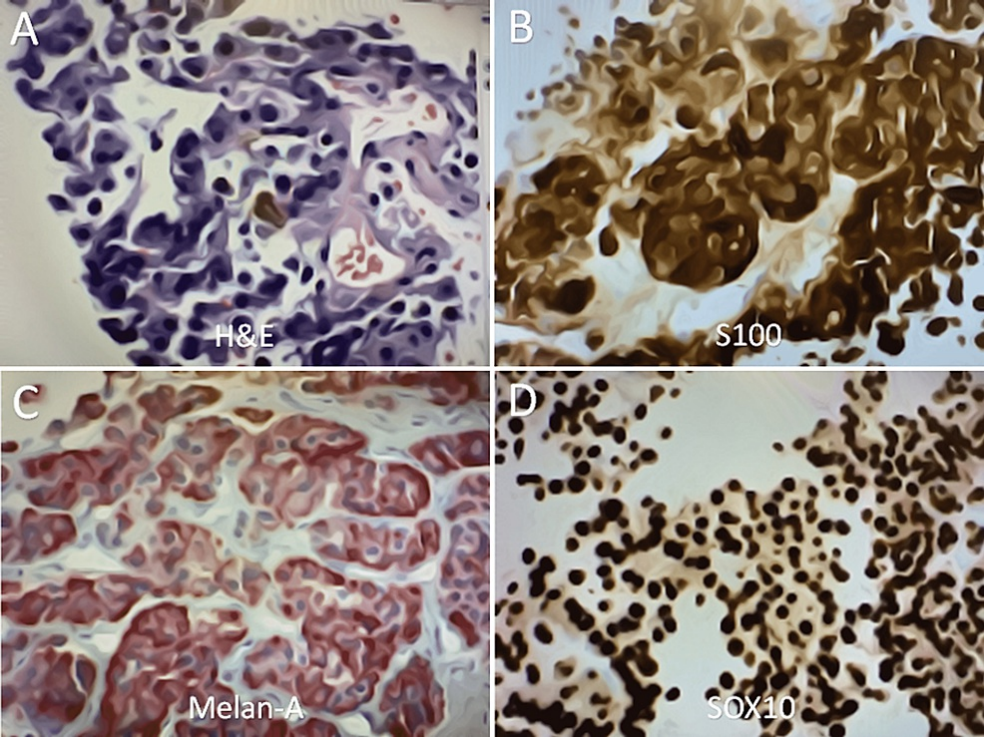
Representative images of an immunostained specimen biopsied from the left adrenal gland. Immunohistochemistry, magnification ×40. (A) H&E, (B) S100 positive, (C) Melan-A positive, and (D) SOX10 positive. H&E: hematoxylin and eosin; SOX10: SRY-related HMG-box protein 10.

Interventional radiology performed a CT-guided biopsy of the left adrenal gland to assist in the diagnosis of this patient. Six days after the biopsy of the left adrenal mass, the pathologist confirmed findings that were consistent with metastatic malignant melanoma, with immunohistochemistry positive for S100, Melan-A, and SRY-related HMG-box protein 10 (SOX10) in the adrenal cortex (Figure 4). Molecular testing of the sample detected a driver mutation of the oncogene BRAF V600E. Cardiology reviewed the CTA of the chest and determined that the cardiac mass was very highly suspicious for metastasis from melanoma to the heart and recommended outpatient evaluation for surgical removal of the mass.

Once the unfortunate results were revealed to the patient, she recalled a melanotic lesion removed from her back over a decade ago. A thorough skin examination performed by two different physicians did not yield any current abnormal or suspicious skin findings consistent with malignant melanoma. Hematology-oncology recommended that the patient should be treated at a tertiary oncology center, where she will start immunotherapy with the ICIs nivolumab plus ipilimumab, undergo further staging imaging with a positron emission tomography (PET) scan and an MRI of the brain, and receive an evaluation for possible surgery. The patient agreed with this plan, and she was counseled on her new diagnosis. She was discharged and kindly allowed for her history and case to be shared with the medical field in the form of this case report.

## Discussion

Metastatic spread of melanoma to the adrenal glands is associated with systemic disease and a poor prognosis [4]. The true incidence of patients with this condition is unknown, as adrenal metastases usually show no symptoms and most cases are discovered incidentally [4]. One study showed up to 50% of patients with stage IV melanoma had adrenal metastases, while other studies have shown a much lower rate of adrenal metastases [4-6]. However, little data exists regarding the natural history of patients with adrenal metastases of malignant melanoma [4].

The exact pathophysiology regarding the predilection of melanoma to infiltrate the adrenal glands is not definitively known. One hypothesis suggests that melanoma cells have a high number of glucocorticoid receptors, so they have a high affinity toward the glucocorticoid-rich adrenal cortex [7]. Indeed, most cases of metastases of melanoma to the adrenal glands involve the adrenal cortex rather than the adrenal medulla, as was seen in our patient [8].

Adrenal metastases from melanoma are initially suspected on imaging, usually with abdominal CT or MRI. Key features suggestive of adrenal metastases are masses that are bilateral, greater than 5 cm in diameter, and have heterogeneous features [5]. All of these features were present in our patient. The diagnosis of adrenal metastases from melanoma is confirmed with specific immunohistochemical stains on histology. Immunohistochemical markers specific to melanoma include the proteins S100, Melan-A, and SOX10 [9]. Patients with adrenal metastases should undergo molecular testing for a driver mutation of the oncogene BRAF V600E, which produces B-Raf, a serine/threonine-protein kinase that plays a critical role in the oncogenesis of melanoma. Patients should also undergo CT of the chest and pelvis, MRI of the brain, and a PET scan to investigate other sites of metastases [10].

One aspect that is essential in diagnosis is differentiating between adrenal metastases from melanoma and primary adrenal melanoma. Both conditions are rare, but primary adrenal melanoma is even less common than adrenal metastases from melanoma. Adrenal metastases tend to be bilateral, are larger in size (>5 cm), and occur in patients with a prior history of melanoma, all of which were present in our patient [11]. On the other hand, primary adrenal melanoma tends to be unilateral, is smaller in size (<5 cm), and occurs in patients without a prior history of melanoma [11]. One definitive way in which adrenal metastases from melanoma can be differentiated from primary adrenal melanoma is by identifying their location in the adrenal gland on histology. As stated above, adrenal metastases from melanoma are usually located in the adrenal cortex [7]. By contrast, primary adrenal melanomas typically originate in the adrenal medulla, as neural crest cells, which serve as both the precursors of melanocytes and chromaffin cells in the adrenal medulla, undergo aberrant morphogenesis during gestation, leading to the presence of melanocytes in the adrenal medulla [11]. In this way, the location of melanoma cells in the adrenal gland (cortex vs. medulla) can be used to differentiate primary adrenal melanoma from metastasis of melanoma to the adrenal glands, as was done in our patient.

The advent of ICIs and BRAF inhibitors has improved the prognosis of metastatic melanoma considerably [1]. The recommended initial therapy for adrenal metastases from melanoma, regardless of BRAF V600E mutation status, is systemic immunotherapy, most commonly with nivolumab plus ipilimumab, which our patient is currently receiving [12]. Other ICI regimens include nivolumab plus relatlimab or single-agent immunotherapy with pembrolizumab [13,14]. If a patient is not a candidate for immunotherapy and is positive for a mutation of BRAF V600E, then combination therapy with a BRAF inhibitor plus mitogen-activated protein kinase (MEK) inhibitor is recommended [15]. The most commonly used regimens are dabrafenib plus trametinib, encorafenib plus binimetinib, or vemurafenib plus cobimetinib. Systemic immunotherapy has shown an improved survival benefit compared to therapy with a BRAF inhibitor plus a MEK inhibitor, even in patients who are positive for a mutation of BRAF V600E [12,15].

Although ICIs have revolutionized the treatment of melanoma, adrenal gland metastases from melanoma are unique in that they remain highly resistant to these new treatments [2,3]. Consequently, patients with metastatic melanoma who have adrenal gland metastases have lower overall survival than those without adrenal gland metastases [2]. It is hypothesized that the reason the adrenal gland is a sanctuary site for metastases is that corticosteroid production creates an immunosuppressive tumor microenvironment. Corticosteroids suppress lymphocyte function and upregulate beta-catenin, which promotes immune cell exclusion from the tumor microenvironment [2]. Furthermore, corticosteroids slow dendritic cell maturation and limit the ability of T cells to recognize tumor cells, which allows tumor cells to evade checkpoint inhibition and proliferate [2]. Other molecules produced in the adrenal gland, such as sex hormones and catecholamines, further suppress the immune system [2]. There have been no studies in the literature evaluating whether adrenal metastases from melanoma demonstrate similar resistance to BRAF and MEK inhibitors in patients who have a mutation of BRAF V600E. This could represent a future area of study, and if no unique resistance to BRAF inhibitors or MEK inhibitors is found, they could serve as a targeted treatment option for adrenal metastases that are positive for a mutation for BRAF V600E.

Surgery remains controversial in the treatment of metastatic melanoma [16]. Although some studies have advocated for an increased role of surgical intervention, many remain skeptical of nonpalliative surgery for metastatic melanoma. For adrenal metastases specifically, a recent review has shown that there is a survival benefit in performing adrenalectomy when metastasis of melanoma is isolated to the adrenal gland and when the site of the primary lesion can be resected [17]. Ultimately, surgical intervention should be discussed by multidisciplinary teams on a case-by-case basis and remains a future area of study.

Our patient has a high clinical suspicion of cardiac metastasis of melanoma, which should be taken into account when forming this patient’s treatment plan. A study showed that 64% of post-mortem melanoma patients had cardiac involvement, but only 2% of patients who were alive had cardiac involvement [18]. Our patient did not have surgery for her right atrial mass while in the hospital, but will have an evaluation for possible surgery of the mass at a tertiary oncology center, at which time a biopsy with pathology could be performed. If confirmed to be melanoma, any surgery on the cardiac mass would be complex, require a long recovery, and would be of questionable survival benefit [16].

In addition, our patient has a high clinical suspicion of tumor thrombus. The vessel expansion seen on imaging suggests that this is a tumor thrombus rather than a bland thrombus [19]. Tumor thrombus would be able to be confirmed by pathologic confirmation, which could be done at her tertiary oncology center. Tumor thrombus is rare in metastatic melanoma, and it is reasonable to hypothesize whether the adrenal metastases contributed to the formation of the thrombus in this patient. Indeed, adrenal tumors, whether primary or metastatic, are one of the most common tumors to form tumor thrombus due to the rich sinusoidal blood supply of the adrenal glands [19]. Venous blood from the adrenal glands drains into the IVC [19]. The spread of melanoma through the adrenal venous supply to the IVC is a possible mechanism by which our patient formed a thrombus in her IVC. It is ultimately difficult to know the exact role adrenal metastases played in the development of thrombus in our patient. Further clinical and molecular studies on more patients need to be done to elucidate whether adrenal metastases in melanoma can contribute to the formation of tumor thrombus.

Our patient’s presentation was complex, and finalizing a treatment plan required extensive discussion among a multidisciplinary team of physicians. Although resistance to ICIs is possible, immunotherapy with nivolumab plus ipilimumab is still the recommended treatment for adrenal gland metastases from melanoma due to a lack of evidence suggesting any other superior option. If our patient does not improve with ICIs, another treatment option would be a BRAF inhibitor plus MEK inhibitor, as the patient has a mutation for BRAF V600E. The patient was recommended to have an outpatient evaluation for surgical removal of her lesions. However, the patient’s disease has likely spread to multiple organs and surgery would be of questionable survival benefit in this case. A recent study showed that percutaneous image-guided thermal ablation of adrenal metastases from melanoma offered favorable local control but did not offer any survival benefit [20]. In our patient with diffuse metastatic disease, she is unlikely to benefit from this therapy. Our case shows how adrenal gland metastases from melanoma are unique in nature and remain clinically challenging.

## Conclusions

Herein, we have discussed a unique case of a patient with previously resected cutaneous melanoma, presenting years later with histologically confirmed metastases to the adrenal glands, as well as suspected metastasis to the heart and a suspected IVC tumor thrombus. This case demonstrates the complexity of metastatic melanoma and the unique ways in which it can present. It highlights the distinguishing features of metastatic melanoma to the adrenals. Ultimately, this case demonstrates the need for further medical research into treatment options for adrenal gland metastases from melanoma, in order to continue to improve management and prognosis.
